# Parameterized hemodynamic response function data of healthy individuals obtained from resting-state functional MRI in a 7T MRI scanner

**DOI:** 10.1016/j.dib.2018.01.003

**Published:** 2018-01-06

**Authors:** D. Rangaprakash, Guo-Rong Wu, Daniele Marinazzo, Xiaoping Hu, Gopikrishna Deshpande

**Affiliations:** a AU MRI Research Center, Department of Electrical and Computer Engineering, Auburn University, Auburn, AL, USA; b Department of Psychiatry and Biobehavioral Sciences, University of California, Los Angeles, Los Angeles, CA, USA; c Department of Data Analysis, University of Ghent, Ghent, Belgium; d Key Laboratory of Cognition and Personality, Southwest University, Chongqing, China; e Department of Bioengineering, University of California Riverside, Riverside, CA, USA; f Department of Psychology, Auburn University, Auburn, AL, USA; g Alabama Advanced Imaging Consortium, Auburn University and University of Alabama Birmingham, AL, USA

## Abstract

Functional magnetic resonance imaging (fMRI), being an indirect measure of brain activity, is mathematically defined as a convolution of the unmeasured latent neural signal and the hemodynamic response function (HRF). The HRF is known to vary across the brain and across individuals, and it is modulated by neural as well as non-neural factors. Three parameters characterize the shape of the HRF, which is obtained by performing deconvolution on resting-state fMRI data: response height, time-to-peak and full-width at half-max. The data provided here, obtained from 47 healthy adults, contains these three HRF parameters at every voxel in the brain, as well as HRF parameters from the default-mode network (DMN). In addition, we have provided functional connectivity (FC) data from the same DMN regions, obtained for two cases: data with deconvolution (HRF variability minimized) and data with no deconvolution (HRF variability corrupted). This would enable researchers to compare regional changes in HRF with corresponding FC differences, to assess the impact of HRF variability on FC. Importantly, the data was obtained in a 7T MRI scanner. While most fMRI studies are conducted at lower field strengths, like 3T, ours is the first study to report HRF data obtained at 7T. FMRI data at ultra-high fields contains larger contributions from small vessels, consequently HRF variability is lower for small vessels at higher field strengths. This implies that findings made from this data would be more conservative than from data acquired at lower fields, such as 3T. Results obtained with this data and further interpretations are available in our recent research study (Rangaprakash et al., in press) [1]. This is a valuable dataset for studying HRF variability in conjunction with FC, and for developing the HRF profile in healthy individuals, which would have direct implications for fMRI data analysis, especially resting-state connectivity modeling. This is the first public HRF data at 7T.

**Specifications Table**

**Table:** 

Subject area	*Brain imaging*
More specific subject area	*Functional magnetic resonance imaging, hemodynamic response variability, hemodynamic response function parameters, ultra-high field MRI, 7T MRI scanner*
Type of data	*Image: brain maps of HRF parameters for every participant*
How data was acquired	*Siemens Magnetom 7T MRI Scanner (Siemens Healthcare, Erlangen, Germany)*
Data format	*NifTi (.nii) and Matlab matrix (.mat)*
Experimental factors	*Our data consisted of a single population of healthy adults from the general society*
Experimental features	*Resting-state: participants kept their eyes open and fixated on a white cross, which was displayed on a dark background, using an Avotec projection system. They were instructed to not dwell on specific thoughts. Each resting-state scan lasted for 11 minutes.*
Data source location	*Auburn, AL, United States of America (GPS coordinates: 32.586, -85.494)*
Data accessibility	*Data has been made available with this article.*

**Value of the data**

• This dataset provides a characterization of the variability of hemodynamic response function (HRF) across the brain, and across individuals, which is a confounding negative factor in functional magnetic resonance imaging (fMRI) data analysis [2], especially connectivity modeling [3].
• This dataset, which also includes comparable functional connectivity (FC) data, is valuable for studying the impact of HRF variability on varieties of fMRI data analyses, including, but not limited to, resting-state FC modeling.
• This dataset characterizes voxel-level HRF variability, hence it could be utilized to develop a generalized whole-brain voxel-level HRF template, with applications in fMRI data analysis.
• This is the first study to present HRF data obtained in a 7T MRI scanner. With less noisy HRF estimates, findings from this dataset would be more conservative than that acquired at lower fields, such as 3T.

## 1 Data

The dataset presented here contains three parameters that characterize the shape of the HRF [3] – response height, time-to-peak and full-width at half-maximum. In the first part of the dataset, each parameter is available at every voxel of the entire brain for every participant, which is provided as 3D NifTi images (*.nii). One image file per parameter per participant is provided. In the second part of the dataset, each HRF parameter is available for the default-mode network (DMN) regions defined by the Power et al. atlas [5], along with corresponding FC between the same regions, thus enabling researchers to compare the two, like in our recent research study [1].

## 2 Experimental design, materials and methods

### 2.1 Participants

Forty-seven healthy adults participated in the study. Resting-state fMRI data was obtained in a 7T MAGNETOM scanner (Siemens Healthcare, Erlangen, Germany) using T2* weighted multiband echo-planar imaging (EPI) sequence [6]. The advantage of data acquisition at 7T is that within-subject HRF variability is likely lower at 7T compared to 3T (thus less noisy), because of larger contributions from smaller vessels [4]. Participants were instructed to have their eyes open, and not contemplate on any specific thoughts. FMRI acquisition parameters were as follows: repetition time (TR)=1000 ms, echo time (TE)=20 ms, flip angle=70°, multiband factor=2, voxel size=2×2×2.4 mm^3^, acquisition matrix=96×96, number of slices=45, and number of fMRI volumes=660 (11 min), with whole-brain coverage. A 32-channel head coil was used. All participants provided informed consent; all procedures were approved by the Auburn University Institutional Review Board (IRB).

### 2.2 FMRI data pre-processing

The following standard pre-processing steps were performed on the resting-state fMRI data: slice-timing correction (since the data was acquired using a multiband sequence), realignment and unwrapping, coregistering to the anatomical image, de-spiking, normalization to the MNI space, spatial smoothing (8 mm Gaussian kernel), and regressing out nuisance covariates (six head-motion parameters, Legendre polynomials of up to second order, top five principle components from participant-specific white matter (WM) signal and cerebrospinal fluid (CSF) signal). Finally, temporal band-pass filtering was performed (0.008–0.1 Hz). Pre-processing was carried out in the Matlab© R2013a platform using Statistical Parametric Mapping (SPM12) [7].

### 2.3 Obtaining the HRF parameters

The voxel-wise 3D+time fMRI data was utilized to perform temporal hemodynamic deconvolution. Latent neural time series and corresponding HRF parameters were obtained at every voxel through this process. For deconvolution, we used a popular technique developed by Wu et al. [8]. The technique has gained increasing popularity and acceptance due to its interpretability, robustness, validity, simplicity of implementation, and an awareness within the research community regarding the need for deconvolution. Several recent studies have utilized it (see for example [9], [10], [11], [12], [13], [14], [15], [16], [17], [18]). Hemodynamic deconvolution is blind because only one variable is accessible (fMRI time series), from which both the latent neural time series and the HRF are estimated. In simple terms, the technique models resting-state fMRI as event-related time series, with randomly occurring events modeled as point processes [19], [20], using which the voxel-wise HRFs are estimated through Wiener deconvolution. The deconvolution code, on the Matlab© platform, is available for download at [21]. A user-interface-based deconvolution toolbox would be released separately in the near future.

Deconvolution provided the estimated HRF at every voxel of the brain, in every participant. It was characterized by three HRF parameters, as noted earlier – response height (RH), time-to-peak (TTP), and full-width at half-max (FWHM) (see Fig. 1 in [3]). The data being made available with this article are these voxel-wise HRF parameters for all the participants. All data analysis was performed on the Matlab® platform.

In addition, we have also provided these three HRF parameters obtained from the DMN, along with the functional connectivity (FC) between corresponding regions. The DMN regions-of-interest (ROIs) were obtained as 10-mm diameter spheres around the DMN centroids as defined in Power et al. [5] (template available with the data). Mean time series were first obtained from the 58 DMN ROIs, and deconvolution was performed on them to obtain latent neural time series and HRF parameters. FC was obtained for all pairwise connections using Pearson's correlation [22], [23]. Our recent research study, using this HRF and FC data [1], assessed the impact of HRF variability on FC, and concluded that HRF variability confounds FC analysis. The implications of those findings are widespread, since most of the resting-state fMRI FC studies (1900+ articles published each year and increasing exponentially) do not perform deconvolution and do not account for HRF variability. This data can be utilized by researchers to compare change in HRF parameters with the corresponding change in FC, using which they could replicate our findings, as well as perform follow-up research and make new discoveries.

Our main findings associated with this dataset, along with further interpretations, are part of our recent research study [1].

## References

[1] D. Rangaprakash, G.-R. Wu, D. Marinazzo, X. Hu and G. Deshpande, Hemodynamic response function (HRF) variability confounds resting-state fMRI functional connectivity Magn. Reson. Med. 2018, in press. /10.1002/mrm.27146 10.1002/mrm.27146 29656446

[2] Handwerker D.A., Ollinger J.M., D'Esposito M. (2004). Variation of BOLD hemodynamic responses across subjects and brain regions and their effects on statistical analyses. Neuroimage.

[3] Rangaprakash D., Dretsch M.N., Yan W., Katz J.S., Denney T.S., Deshpande G. (2017). Hemodynamic variability in soldiers with trauma: implications for functional MRI connectivity studies. NeuroImage: Clin..

[4] Yacoub E., Shmuel A., Pfeuffer J., Van De Moortele P.F., Adriany G., Andersen P., Vaughan J.T., Merkle H., Ugurbil K., Hi X. (2001). Imaging brain function in humans at 7 T. Magn. Reson. Med..

[5] Power J.D., Cohen A.L., Nelson S.M., Wig G.S., Barnes K.A. (2011). Functional network organization of the human brain. Neuron.

[6] Feinberg D.A., Moeller S., Smith S.M., Auerbach E. (2010). Multiplexed Echo Planar Imaging for Sub-Second Whole Brain FMRI and Fast Diffusion Imaging. PLoS One.

[7] Friston K.J., Ashburner J., Kiebel S.J., Nichols T.E., Penny W.D. (2007). Statistical Parametric Mapping: The Analysis of Functional Brain Images.

[8] Wu G., Liao W., Stramaglia S., Ding J., Chen H., Marinazzo D. (2013). A blind deconvolution approach to recover effective connectivity brain networks from resting state fMRI data. Med Image Anal..

[9] Boly M., Sasai S., Gosseries O., Oizumi M., Casali A., Massimini M., Tononi G. (2015). Stimulus set meaningfulness and neurophysiological differentiation: a functional magnetic resonance imaging study. PLoS One.

[10] Rangaprakash D., Deshpande G., Daniel T., Goodman A., Robinson J., Salibi N., Katz J., Denney T., Dretsch M. (2017). Compromised Hippocampus-Striatum Pathway as a Potential Imaging Biomarker of Mild Traumatic Brain Injury and Posttraumatic Stress Disorder. Hum. Brain Mapp..

[11] Lamichhane B., Adhikari B.M., Brosnan S.F., Dhamala M. (2014). The neural basis of perceived unfairness in economic exchanges. Brain Connect..

[12] Rangaprakash D., Dretsch M., Venkatraman A., Katz J., Denney T., Deshpande G. (2018). Identifying Disease Foci from Static and Dynamic Effective Connectivity Networks: illustration in Soldiers with Trauma. Hum. Brain Mapp..

[13] Amico E., Gomez F., Di Perri C., Vanhaudenhuyse A., Lesenfants D., Boveroux P., Bonhomme V., Brichant J.F., Marinazzo D., Laureys S. (2014). Posterior cingulate cortex-related co-activation patterns: a resting state FMRI study in propofol-induced loss of consciousness. PLoS One.

[14] R. Tadayonnejad, R. Deshpande, O. Ajilore, T. Moody, F. Morfini, R. Ly, J. O'Neill and J. Feusner, Pregenual anterior cingulate dysfunction associated with depression in OCD: an integrated multimodal fMRI/1H MRS study, Neuropsychopharmacology 2017, in press. 10.1038/npp.2017.249 10.1038/npp.2017.249 PMC5854805 29052616

[15] Rangaprakash D., Dretsch M.N., Yan W., Katz J.S., Denney T.S., Deshpande G. (2017). Hemodynamic response function parameters obtained from resting-state functional MRI data in soldiers with trauma. Data Brief.

[16] Zhao S., Rangaprakash D., Venkataraman A., Liang P., Deshpande G. (2017). Investigating Focal Connectivity Deficits in Alzheimer's Disease Using Directional Brain Networks Derived from Resting-State fMRI. Front. Aging Neurosci..

[17] Jin C., Jia H., Lanka P., Rangaprakash D., Li L., Liu T., Hu X., Deshpande G. (2017). Dynamic brain connectivity is a better predictor of PTSD than static connectivity. Hum. Brain Mapp..

[18] Rangaprakash D., Dretsch M., Katz J., Denney T., Deshpande G. (2018). Dynamics of Segregation and Integration in Directional Brain Networks: illustration in Soldiers with PTSD and Neurotrauma. Neuroimage: Clin..

[19] Saad Z.S., Gotts S.J., Murphy K., Chen G., Jo H.J., Martin A., Cox R.W. (2012). Trouble at rest: how correlation patterns and group differences become distorted after global signal regression. Brain Connect..

[20] Power J.D., Schlaggar B.L., Petersen S.E. (2015). Recent progress and outstanding issues in motion correction in resting state fMRI. Neuroimage.

[21] D. Marinazzo, Code for HRF blind deconvolution, [Online]. Available: 〈http://users.ugent.be/~dmarinaz/HRF_deconvolution.html〉. (Accessed September 2016).

[22] Rangaprakash D. (2017). Binarized Brain Connectivity: a Novel Autocorrelation-Based Iterative Synchronization Technique. IEEE Trans. Signal Inf. Process. Netw..

[23] Rangaprakash D., Hu X., Deshpande G. (2013). Phase synchronization in brain networks derived from correlation between probabilities of recurrences in functional MRI data. Int. J. Neural Syst..

