# Supplementary material for: Parameterized hemodynamic response function data of healthy individuals obtained from resting-state functional MRI in a 7T MRI scanner
Source: Data Brief. 2018 Jan 6;17:1175–9. doi: 10.1016/j.dib.2018.01.003 (PMC5988211; doi:10.1016/j.dib.2018.01.003)
Supplement: Supplementary file 1 — Supplementary material [file mmc1.zip › HRF_parameters_Healthy_7T_Auburn/Readme.pdf]

Folder Structure

<u>Legend:</u>

DMN = default-mode network

FC = functional connectivity

FWHM = full-width at half-maximum HRF parameter

RH = response height HRF parameter

TTP = time-to-peak HRF parameter

_sub_xx = subject number

<u>The folder structure-</u>

<u>(text in green is a folder, text in red is a file and text in blue is its description):</u>

- Power-264 template data
  - → *FunctionalConnectivity_Power264_DMN.mat*

    Functional connectivity (FC) data provided for two cases (file can be loaded in Matlab):
    - DC = using resting-state fMRI data with Deconvolution performed (HRF-variability-minimized)
    - NDC = using resting-state fMRI data with No Deconvolution (HRF-variability-ignored)

    Each of the two variables has dimensions 58x58x47, where 58x58 is the FC matrix obtained between all pairs of the 58 DMN ROIs, and 47 are the subjects.

  - → *HRF_parameters_Power264_DMN.mat*

    Each of the three HRF parameters is a separate variable
    Each variable has dimensions 58x47
    ...where 58 are the DMN ROIs and 47 are the subjects

  - → *Power264_template_DMN.mat*

    Power et. al. (Neuron, 2011) 264 regions functional template: this table provides information about the 58 default mode network regions.

- Whole-brain HRF data

  Here, each NifTi file contains voxel-level values of the corresponding HRF parameter. Repetition time (TR) = 1000ms.

  → FullWidthHalfMax
    → *FWHM_sub_xx.nii* (47 files)

  → ResponseHeight
    → *RH_sub_xx.nii* (47 files)

  → TimeToPeak
    → *TTP_sub_xx.nii* (47 files)

×××××××
