# Supplementary figures and images for: Parameterized hemodynamic response function data of healthy individuals obtained from resting-state functional MRI in a 7T MRI scanner
Source: Data Brief. 2018 Jan 6;17:1175–9. doi: 10.1016/j.dib.2018.01.003 (PMC5988211; doi:10.1016/j.dib.2018.01.003)

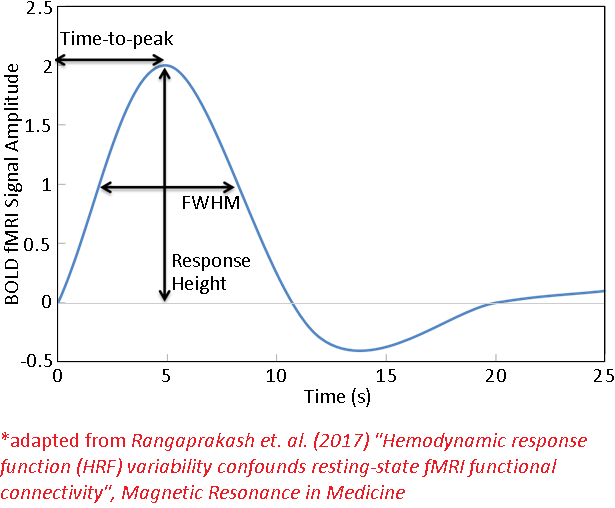

Supplement: Supplementary file 1 — Supplementary material [file mmc1.zip › HRF_parameters_Healthy_7T_Auburn/HRFparameters_illustration.tif]

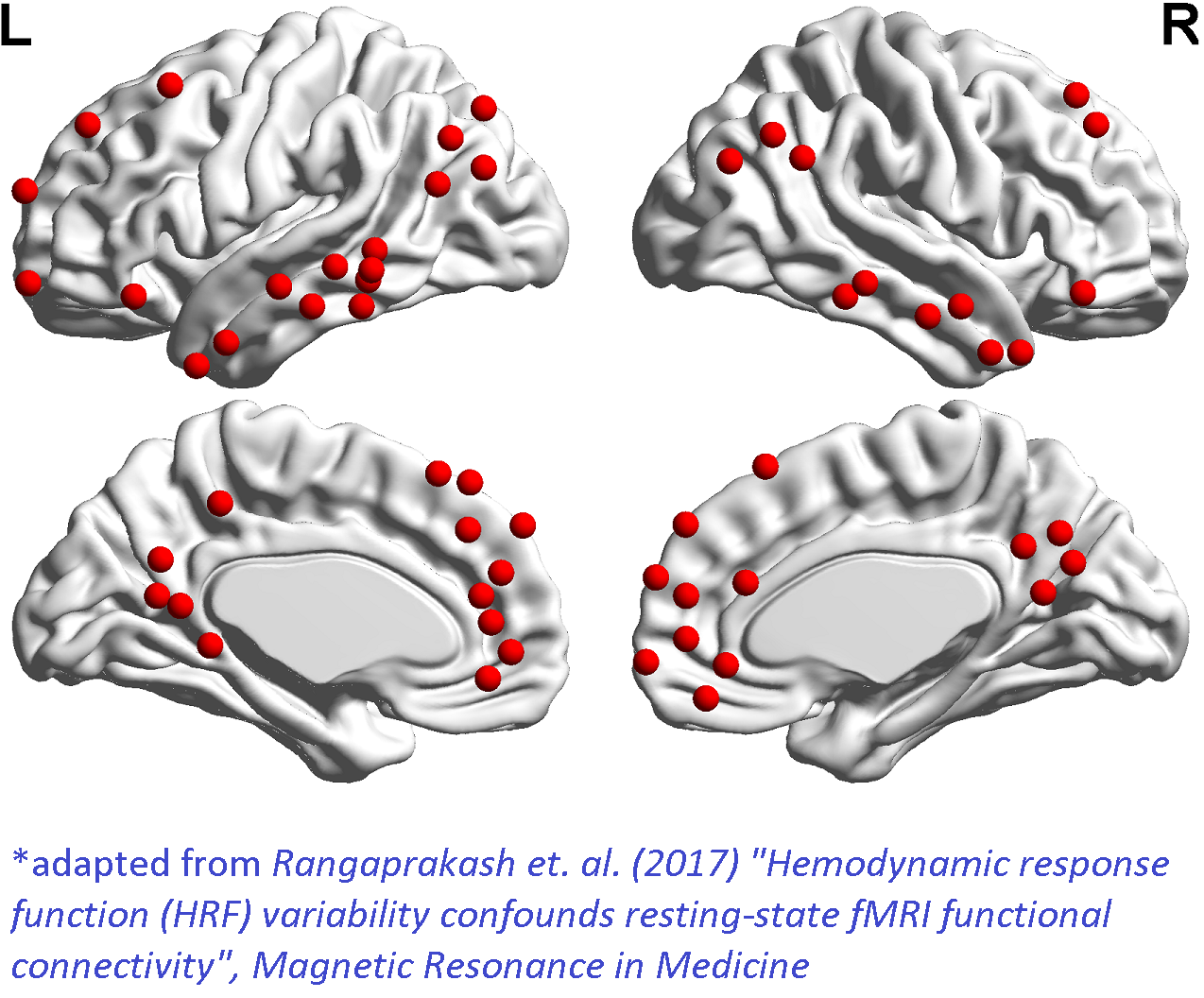

Supplement: Supplementary file 1 — Supplementary material [file mmc1.zip › HRF_parameters_Healthy_7T_Auburn/Power-264 template data/Power264_DMN_ROIs.tif]
